# Supplementary material for: Identifying repeat domains in large genomes
Source: Genome Biol. 2006 Jan 31;7(1):R7. doi: 10.1186/gb-2006-7-1-r7 (PMC1431705; doi:10.1186/gb-2006-7-1-r7)
Supplement: Additional File 1 — A zipped file of browsable HTML files with a complete list of the connected components in the repeat domain graph of human Repbase. [file gb-2006-7-1-r7-S1.gz › html/subgraphs/8024.html]

|  |  |
| --- | --- |
| id | repbase name |
| 21 | THE1BR |
| 22 | MSTAR |
| 23 | MLT1R |
| 492 | MLT1AR |
| 493 | MLT1CR |
| 571 | MLT1FR |
